# Calreticulin is required for calcium homeostasis and proper pollen tube tip growth in *Petunia*

**DOI:** 10.1007/s00425-017-2649-0

**Published:** 2017-01-11

**Authors:** Anna Suwińska, Piotr Wasąg, Przemysław Zakrzewski, Marta Lenartowska, Robert Lenartowski

**Affiliations:** 1 Laboratory of Developmental Biology, Faculty of Biology and Environmental Protection, Nicolaus Copernicus University in Toruń, Toruń, Poland; 2 Laboratory of Isotope and Instrumental Analysis, Faculty of Biology and Environmental Protection, Nicolaus Copernicus University in Toruń, Toruń, Poland

**Keywords:** Actin cytoskeleton, Calcium gradient, Cytoplasmic zonation, In vitro studies, Small interfering RNA, Ultrastructural research

## Abstract

**Calreticulin is involved in stabilization of the tip-focused Ca** ^**2+**^ **gradient and the actin cytoskeleton arrangement and function that is required for several key processes driving** ***Petunia*** **pollen tube tip growth.**

Although the precise mechanism is unclear, stabilization of a tip-focused calcium (Ca^2+^) gradient seems to be critical for pollen germination and pollen tube growth. We hypothesize that calreticulin (CRT), a Ca^2+^-binding/buffering chaperone typically residing in the lumen of the endoplasmic reticulum (ER) of eukaryotic cells, is an excellent candidate to fulfill this role. We previously showed that in *Petunia* pollen tubes growing in vitro, CRT is translated on ER membrane-bound ribosomes that are abundant in the subapical zone of the tube, where CRT’s Ca^2+^-buffering and chaperone activities might be particularly required. Here, we sought to determine the function of CRT using small interfering RNA (siRNA) to, for the first time in pollen tubes growing in vitro, knockdown expression of a gene. We demonstrate that siRNA-mediated post-transcriptional silencing of *Petunia hybrida CRT* gene (*PhCRT*) expression strongly impairs pollen tube growth, cytoplasmic zonation, actin cytoskeleton organization, and the tip-focused Ca^2+^ gradient. Moreover, reduction of CRT alters the localization and disturbs the structure of the ER in abnormally elongating pollen tubes. Finally, cytoplasmic streaming is inhibited, and most of the pollen tubes rupture. Our data clearly show an interplay between CRT, Ca^2+^ gradient, actin-dependent cytoplasmic streaming, organelle positioning, and vesicle trafficking during pollen tube elongation. Thus, we suggest that CRT functions in *Petunia* pollen tube growth by stabilizing Ca^2+^ homeostasis and acting as a chaperone to assure quality control of glycoproteins passing through the ER.

**Electronic supplementary material:**

The online version of this article (doi:10.1007/s00425-017-2649-0) contains supplementary material, which is available to authorized users.

## Introduction

In angiosperms, sexual reproduction requires that pollen hydrates on the stigma and then elongates a pollen tube that delivers two immotile sperm cells to the ovule for double fertilization. Pollen tube growth occurs only at the tip via exocytosis at the tube apex. Thus, the elongated pollen tube has a highly polarized cytoplasm comprising four structurally and functionally distinct zones (see reviews by Guan et al. 2013; Hepler et al. 2013; Cai et al. 2015; Hepler and Winship 2015). The apical zone (also known as the clear zone) contains secretory and endocytic vesicles and very few mitochondria or dictyosomes. Golgi-derived vesicles fuse with the apical plasma membrane to provide cell wall and membrane extensions for rapid pollen tube growth. Just proximal to the apical clear zone, the subapical region contains abundant rough ER (rER) and smooth ER (sER), Golgi stacks, mitochondria, plastids, and a few small vacuoles and lipid bodies. Proximal to the subapical zone, the distal shank contains the male germ unit (MGU) and numerous large organelles, such as starch-rich amyloplasts and small vacuoles; abundant rER and some mitochondria or dictyosomes are also observed in this region. Finally, the proximal shank of the elongated pollen tube is full of large vacuoles and a few metabolically active organelles in the residual cytoplasm along the cell periphery. This vacuolated zone extends toward the pollen grain and, as the pollen tube grows, callose plugs isolate this domain from the distal cytoplasmic regions.

This polarized cytoplasm provides a structural basis for pollen tube tip growth and is thought to depend on different actin organization in distinct pollen tube zones (see reviews by Fu 2010; Hepler et al. 2013; Cai et al. 2015; Qu et al. 2015). In the proximal and distal shank, long actin filaments (AFs) are arranged into stable bundles that extend longitudinally through the pollen tube cytoplasm but do not penetrate into the growing domain. These actin cables exhibit uniform polarity, but they are oriented in opposite directions in the periphery and in the central regions of the elongating tube (Lenartowska and Michalska 2008). This organization and the action of the plus-end-directed actin motor myosin XI generate a bidirectional reverse-fountain pattern of cytoplasmic streaming, which carries organelles and vesicles between the shank and the tip of elongating pollen tube (see review by Yamamoto 2008). In the growing domain (subapical and apical zones), highly dynamic filamentous actin (F-actin) structures provide actin tracks for membrane-targeted vesicular trafficking to the apical dome. In the sub-apex, short AFs form a cortical structure described in different plant species as a collar, ring, mesh, funnel, or actin fringe (see review by Qu et al. 2015). In *Arabidopsis*, mutations that disrupt this structure also disrupt pollen tube growth (Dong et al. 2012). Most of the organelles normally stop at this region and/or reverse their motion along the curved subapical actin bundles to the base of the tube. However, secretory vesicles are able to cross the actin fringe and accumulate in the apical cytoplasm to finally reach the apical membrane. The apex of the elongating pollen tube contains an extremely dynamic meshwork of randomly oriented short AFs. This tip-localized population of AFs has been investigated in pollen tubes of a few species including *Arabidopsis*, *Lilium*, *Nicotiana* (see review by Qu et al. 2015), *Haemanthus* (Lenartowska and Michalska 2008), and *Petunia* (this paper). Together with the actin fringe, these AFs are thought to organize docking and fusion of secretory vesicles and endomembrane trafficking events. Thus, highly ordered AFs (especially in the shank) and highly dynamic AFs (especially in the growing domain) are fundamental components of polar growth in angiosperm pollen tubes. However, how these diverse actin structures are properly generated and maintained remains poorly understood.

Different aspects of actin dynamics and architecture in growing pollen tubes are regulated by several classes of actin-binding proteins (ABPs) such as profilin, ADF/cofilin, villin, gelsolin, and formin. Each of these proteins carries out specific functions by interacting with monomeric actin (G-actin) and/or F-actin. Most of these proteins are tightly modulated by different cellular parameters including pH, phosphoinositides, phosphorylation, and protein–protein interactions. Intracellular Ca^2+^ also plays a crucial role in regulating both ABPs and pollen tube growth (see reviews by Ren and Xiang 2007; Staiger et al. 2010; Qu et al. 2015). The growing pollen tube possesses a steep tip-focused gradient of cytosolic Ca^2+^ that is created by Ca^2+^ influx at the apex and is closely correlated with growth rate and morphology of the elongating tube (see reviews by Holdaway-Clarke and Hepler 2003; Hepler et al. 2011; Steinhorst and Kudla 2013). Thus, even though some ABPs are uniformly distributed throughout the pollen tube cytoplasm, their activity can be regulated by the local cytosolic Ca^2+^ concentration ([Ca^2+^]_c_) to differentially affect actin assembly in each zone of the tube. Therefore, Ca^2+^ homeostasis appears to be critical for proper actin organization and function and, consequently, establishment and maintenance of pollen tube tip growth.

We previously suggested that the key molecule controlling Ca^2+^ gradient in growing *Petunia* pollen tubes is the Ca^2+^-binding protein CRT, which is translated on membrane-bound ribosomes and accumulates in the abundant ER in the subapical zone of the tube (Suwińska et al. 2015). In this region, the high [Ca^2+^]_c_ at the apex drops off sharply to the basal level observed throughout the shank (see reviews by Holdaway-Clarke and Hepler 2003; Hepler et al. 2011; Steinhorst and Kudla 2013). Here, we further tested this hypothesis using siRNA to selectively degrade *PhCRT* mRNA in *Petunia* pollen tubes growing in vitro. Although another tip-growing cell type, germinated *Aspergillus* spores, has been shown to take up siRNA from the culture medium (Khatri and Rajam 2007), this is the first demonstration of this technique in elongating pollen tubes. Our data provide evidence that CRT expression is required for maintenance of the tip-focused Ca^2+^ gradient and proper pollen tube actin organization and function. We suggest that CRT plays a crucial role in stabilizing the Ca^2+^ homeostasis that is required for several interdependent processes driving *Petunia* pollen tube elongation: acto-myosin-dependent cytoplasmic streaming, organelle positioning, vesicle trafficking, and cell wall biogenesis.

## Materials and methods

### Plant material and pollen tube cultures

Freshly collected mature pollen of *Petunia hybrida* (commercial cultivars grown at room temperature) was germinated in liquid culture medium containing 0.2% sucrose, 0.05% Ca(NO)_3_, 0.01% MgSO_4_, 0.01% H_3_BO_4_, 0.01% KNO_3_, 15% polyethylene glycol 4000, 0.4% 2-(*N*-morpholino)ethanesulfonic acid, pH 6.0. In post-transcriptional gene silencing (PTGS) experiments, the culture medium was supplemented with (1) *PhCRT*-specific siRNA, (2) scrambled siRNA (scrRNA, negative control), or (3) single-stranded DNA oligonucleotides (ssDNA, control for potential oligonucleotide-induced cytotoxicity), and the cultures were incubated at 30 °C for about 4–5 h. Pollen tubes growing in either the standard medium or medium supplemented with siRNA, scrRNA, or ssDNA were prepared for detailed morphological observations using light microscopy, fluorescent in situ hybridization (FISH), immunolabeling, immunoblotting, actin staining, electron microscopy, and intracellular Ca^2+^ detection as described below. For each experiment, pollen collected from a few flowers was mixed in the following ratio: pollen grains from five anthers/3 ml liquid culture medium. All experiments were repeated at least three times with similar results.

### siRNA/scrRNA and ssDNA oligonucleotides used in PTGS experiments

The full-length *PhCRT* cDNA sequence (accession number HG738129, Lenartowski et al. 2014) was analyzed with SVM siRNA Design Tool software (Applied Biosystem, Thermo Fisher Scientific). Predicted target regions found in a BLAST search (http://blast.ncbi.nlm.nih.gov/Blast.cgi) to contain more than twelve contiguous bases pairs of homology to other *Petunia* genes in the NCBI database were excluded from further analysis. Three different antisense siRNA sequences: (si1) 5′AACUGGAAUACUAAGGUUU3′, (si2) 5′GAUCAUCGAUAUAUUCCUU3′, and (si3) 5′ACACCCACAUACUUCAGAU3′ were determined in SSEARCH (http://www.ebi.ac.uk/services/dna-rna) to be specific to *PhCRT* mRNA. The seed regions of each siRNA were aligned with sequences in a small RNA library from *Petunia* floral bud RNA (http://www.bioinformatics.leeds.ac.uk/petunia). Sequences without similarity to *Petunia* small RNA were used as a template for siRNA synthesis. Additionally, siRNAs were confirmed to not include tracts of more than four consecutive homonucleotides and to have a G/C content between 28.6 and 38.1%. A scrambled version of si1*PhCRT* that did not target any gene was used as the negative control (scr_si1*PhCRT* 5′UUGUAGACUUAUAGAAAGC3′). Each oligo was modified at the 3′ end with two [dT] nucleotides to increase nuclease stability (Elbashir et al. 2001) and delivered in duplex format (sense and antisense strands) by Sigma-Aldrich. The sequence of the ssDNA oligo (used as an additional control) was 5′AACTGGAATACTAAGGTTT3′ (Genomed). All the above RNA/DNA sequences were added separately to the culture media at the indicated final concentrations (see “Results”), and then pollen tubes were cultivated up to 4–5 h.

### Light and transmission electron microscopy of in vitro growing pollen tubes

Cultivated control (wild type, WT) and si1*PhCRT*-, scr_si1*PhCRT*-, and ssDNA-treated pollen tubes were fixed with 2% glutaraldehyde (Sigma-Aldrich) in 0.1 M phosphate-buffered saline (PBS), pH 7.2, for 2 h at room temperature. Next, samples were washed three times in PBS buffer, placed onto microscope slides, and observed under the light microscope. For callose imaging, WT, si1*PhCRT*, and scr_si1*PhCRT* pollen tubes were stained with 0.1% aniline blue according to the standard protocol. To identify morphological defects caused by PTGS, pollen tubes were fixed every half hour during the 5-h culture and then examined by light and fluorescence microscopy. Images were acquired using an Olympus BX50 optical/fluorescence microscope, Olympus XC50 color camera, and Cell^B^ software (Olympus Soft Imaging GmbH).

For detailed ultrastructural analysis, elongated WT and si1*PhCRT* pollen tubes (cultivated for about 4–5 h) were fixed and prepared for transmission electron microscopy as previously described (Suwińska et al. 2015). In brief, pollen tubes were fixed with 2% glutaraldehyde (Sigma-Aldrich) in 0.1 M PBS, pH 7.2, for 2 h at room temperature followed by overnight at 4 °C, and then post-fixed with 2% osmium tetroxide (Sigma-Aldrich) in PBS for 30 min at room temperature. Samples were rinsed with PBS and Milli-Q-filtered water, dehydrated in ethanol, and embedded in Poly/Bed 812 resin (Polysciences) according to the standard protocol. Ultrathin longitudinal sections of elongated pollen tubes were post-stained with uranyl acetate and lead citrate solutions before observation by a Joel EM 1010 transmission electron microscope.

### FISH of *PhCRT* mRNA and immunolocalization of CRT in cultivated pollen tubes

Immunolocalization and FISH of CRT and its transcripts in pollen tubes were performed exactly according to the protocols described previously (Suwińska et al. 2015). In brief, for FISH, elongated WT, si1*PhCRT*, and scr_si1*PhCRT* pollen tubes (cultivated for about 4–5 h) were fixed in freshly prepared 4% formaldehyde (Polysciences) in PBS, pH 7.2, enzymatically digested, and permeabilized with 0.1% saponin. CRT transcripts were localized with a digoxigenin (DIG)-labeled antisense *PhCRT* RNA molecular probe (Lenartowski et al. 2014). Pre-hybridization and hybridization were carried out in 50% formamide, 4× SSC, 5× Denhardt’s, 1 mM EDTA, and 50 mM sodium phosphate buffer, pH 7.0. Hybridization signals were detected with an anti-DIG-Rhodamine antibody (Roche), and a no-probe negative control was also performed. To detect CRT protein, pollen tubes were fixed with freshly prepared 4% formaldehyde (Polysciences) in PBS, pH 7.2 and permeabilized with 0.1% Triton X-100. After blocking in 3% bovine serum albumin (Sigma-Aldrich), samples were incubated with a rabbit polyclonal antibody against maize CRT (CRT PAb, Napier et al. 1995) and then with goat anti-rabbit IgG Cy3^®^ secondary antibody (Sigma-Aldrich). A negative control was performed by omitting the primary antibody; the specificity of the CRT PAb was previously verified by immunoblot (Lenartowski et al. 2015; Suwińska et al. 2015). Specimens were covered with MobiGLOW mounting medium (MoBiTec) to prolong the fluorescence. Images of FISH and immunolocalization were acquired using the software package EZ 2000 Viewer connected to a fluorescence inverted Nikon confocal microscope (PCM 2000-Eclipse TE 300) with a 40× (numerical aperture, 0.95) Plan Apochromat objective.

### Western blotting

In vitro elongated WT and si1*PhCRT* pollen tubes (cultivated about 4–5 h) were collected from the culture medium and homogenized in liquid nitrogen, and soluble proteins were extracted in 100 mM Tris–HCl, pH 7.5, 10% sucrose, 5 mM EGTA, 5 mM EDTA, 2 mM DTT, and Complete Protease Inhibitor Cocktail (Roche) according to the manufacturer’s recommendation. The homogenates were centrifuged at 16,000 *g* for 30 min at 4 °C. Protein concentrations of the supernatants were measured with the Bio-Rad DC Protein Assay according to the manufacturer’s instructions. Equal amounts of protein were separated by electrophoresis on a 12.0% SDS–PAGE gel and then the proteins were semi-dry transferred to Amersham PVDF Hybond-P membrane (GE Healthcare). Blocked blots were probed with the CRT PAb or with a mouse anti-actin monoclonal antibody (JLA20, Calbiochem), washed, and probed with anti-rabbit IgG or anti-mouse IgG/IgM secondary antibodies conjugated with horseradish peroxidase (HRP, Sigma-Aldrich and Merck, respectively). Signals were detected with the Amersham ECL Advance Western Blotting Detection Kit according to the manufacturer’s guidelines (GE Healthcare). Subsequently, membranes were stripped and re-probed with a mouse anti-α-tubulin monoclonal antibody (Thermo Fisher Scientific) and then with anti-mouse IgG-HRP secondary antibody (Sigma-Aldrich). Detection was performed as described above. Each western blot experiment was performed five times, and representative blots are shown. Signals were quantified with Image Gauge 3.4 software (Science Lab99), and statistical significance of obtained data was determined by the Mann–Whitney test.

### F-actin staining in growing pollen tubes

In vitro elongated WT and si1*PhCRT* pollen tubes (cultivated about 4–5 h) were transferred to liquid culture medium containing 400 μM *m*-maleimidobenzoyl-*N*-hydroxysuccinimide ester (Sigma-Aldrich) for 6 min. Next, pollen tubes were permeabilized with an actin-stabilizing buffer (ASB) composed of 100 mM PIPES, 5 mM MgSO_4_, 0.5 mM CaCl_2_, and 0.05% Triton X-100, pH 9.0, for 5 min, and fixed with freshly prepared 2% formaldehyde in ASB at room temperature for 30 min. Pollen tubes were washed three times with ASB as above except at pH 7.0 and supplemented with 10 mM EGTA (Sigma-Aldrich). After washing, the samples were labeled with 1 μM AlexaFluor^®^488 Phalloidin (Molecular Probes) ASB-EGTA without Triton X-100 for 30 min in the dark. Finally, labeled pollen tubes were transferred to microscope slides and covered with MobiGLOW mounting medium and images of microfilaments were acquired using the software package LAS AF connected to a Leica SP8 confocal microscope with a 63× (numerical aperture, 1.4) Plan Apochromat DIC H immersion oil lens.

### Intracellular Ca^2+^ detection

Detection of Ca^2+^ in elongated WT and si1*PhCRT* pollen tubes (cultivated about 4–5 h) was performed, with slight modifications, according to a new method (Qu et al. 2016) of loading fluo-4 acetoxymethyl ester (fluo-4/AM) into tip-growing plant cells, such as pollen tubes and root hairs. According to the protocol, 50 µg of fluo-4/AM (Life Technologies) was dissolved in 91.2 µl of anhydrous dimethylsulfoxide (DMSO) in a microcentrifuge tube and diluted with standard culture medium to a 100 µM fluo-4/AM working solution. Cell lysis solution containing 2% cetyltrimethylammonium bromide (CTAB), 100 mM Tris–HCl, and 40 mM EDTA (pH 8.0) was diluted 20-fold with Milli-Q-filtered water. Next, exactly 1 µl of the fluo-4/AM working solution and 1 µl of the cell lysis solution were mixed and added to the individual centrifuge tubes containing 48 µl of the in vitro elongating WT or si1*PhCRT* pollen tubes and incubated in the dark for 15 min at 25 °C. Next, the pollen tubes were washed three times with dye-free basic culture medium and transferred to microscope slides. The yellowish green fluorescence (~516 nm) of the fluo-4/AM-Ca^2+^ was then detected by confocal microscopy as described above.

### Quantitative measurements of fluorescence and statistical analysis

For quantitative measurements, each experiment was performed with consistent experimental conditions and concentrations of the molecular probe, primary and secondary antibodies, AlexaFluor^®^488 Phalloidin, and fluo-4/AM. For FISH and CRT immunolabeling, a small pinhole and 75 µs exposure time were used, For Ca^2+^ detection and F-actin staining, a 95.5 µm pinhole and 400 or 200 kHz exposure time (respectively) were used for all analyzed samples. Three-dimensional optical sections of pollen tubes were acquired with a 1.0 µm (FISH and CRT immunofluorescence images) or 0.5 µm (F-actin and Ca^2+^ detection images) step intervals, from a minimum of 20 comparable pollen tubes during each of the experimental conditions. All data were corrected for background autofluorescence as determined by signal intensities in negative controls. For image processing and analysis, the EZ Viewer software package (Nikon Europe BV) and ImageJ (NIH, Bethesda, MD, USA) software were used. For signal evaluation, Cell Statistical Analyser and ImageGauge 3.46 Fujifilm software were used. The total fluorescence intensity was measured per single pollen tube. PAST 3 software and Microsoft Excel (Microsoft) were used for statistical analysis, and statistical significances of all data were determined by a one-way ANOVA or the Mann–Whitney tests, as appropriate.

## Results

### Knockdown of *PhCRT* expression during pollen tube elongation

To reduce *PhCRT* expression in pollen tubes growing in vitro, we used siRNA SVM Design Tool software (Wang et al. 2009a) to predict functional siRNA candidates in the *PhCRT* cDNA sequence. See “Materials and methods” for a full description of siRNA sequence identification methods used to design three *PhCRT*-specific siRNA oligonucleotides: si1*PhCRT* (+320/+338), si2*PhCRT* (+718/736), and si3*PhCRT* (+986/+1004). As an initial test of these three siRNAs, we supplemented pollen tube culture medium with few various concentrations of siRNA duplexes (from 1.5 to 15 nM) and growing pollen tubes have been observed at different time points (every 30 min). Preliminary observations using stereo/light microscopy revealed that of the three tested siRNAs, only si1*PhCRT* efficiently affected pollen tube growth, giving the same phenotypes of elongating pollen tubes at the all concentrations tested; si2*PhCRT* had no effect, and si3*PhCRT* exerted similar effect as si1*PhCRT*, but at higher concentrations than 1.5 nM (data not shown). It should be noted that the aforementioned outcomes can be observed during the PTGS experiments as a result of different activity of siRNAs designed to knockdown the same mRNA sequence; they can hybridize to highly structured region or polymorphic site in the mRNA or have different thermo-dynamic properties determined by G/C content. Degradation efficiency depends also on the concentration of siRNA and the lowest working concentration should be experimentally determined to maximize the specificity of RNAi effect. Therefore, we finally used si1*PhCRT* at the lowest concentration (1.5 nM) that affected *Petunia* pollen tube growth as the most effective siRNA duplex in all subsequent PTSG experiments. As we were concerned about off-target effects, we confirmed that si1*PhCRT* did not share a seed sequence with any endogenous micro RNA sequences deposited in the *Petunia* small RNA library.

**Fig. 1:**
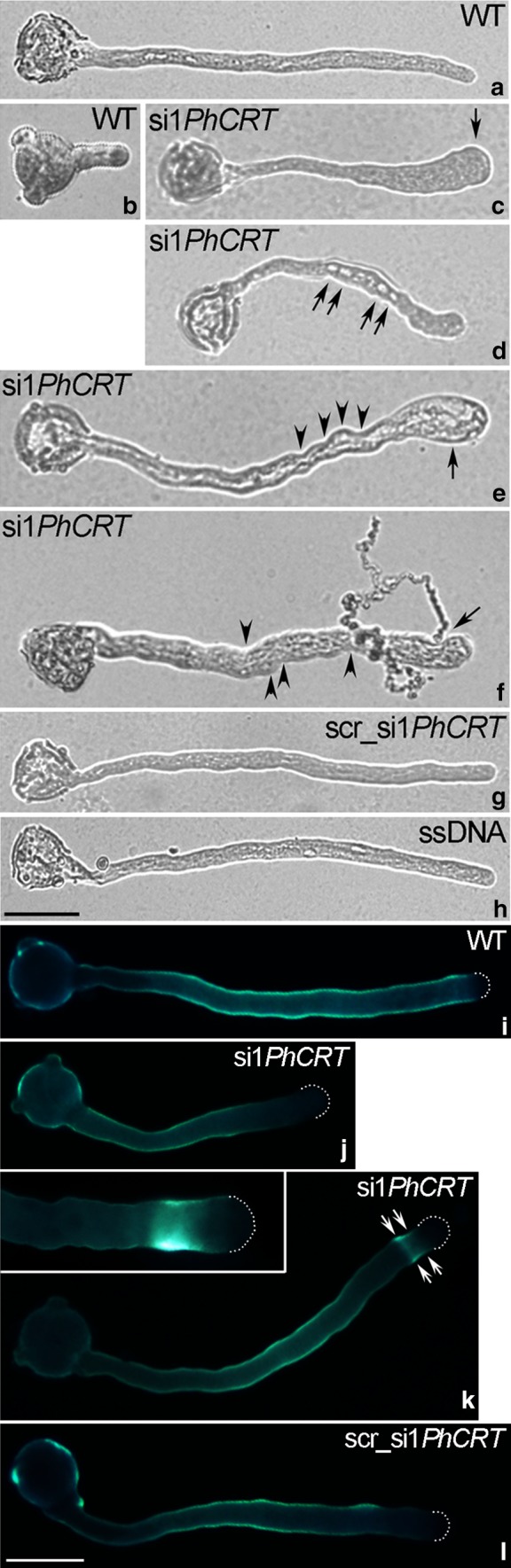
Morphology (**a**–**h**) and callose deposition (**i**–**l**) of/in *Petunia* WT (**a**, **i**), si1*PhCRT* (**c**–**f**, **j**, **k**), scr_si1*PhCRT* (**g**, **l**), and ssDNA (**h**) elongating pollen tubes. **b** Outgrowing pollen tube, the stage when si1*PhCRT* duplex was added to the standard culture medium. **c** Swollen tip of shorter si1*PhCRT* pollen tube (*arrow*). **d** Transparent elements in the cytoplasm of shorter si1*PhCRT* pollen tube (*arrows*). **e** Widely and highly vacuolated tip (*arrow*), and strongly twisted/highly vacuolated distal shank (*arrowheads*) of si1*PhCRT* extended pollen tube. **f** Twisted shank (*arrowheads*) and rupture of the membrane/cell wall at the growing domain (*arrow*) of si1*PhCRT* fully elongated pollen tube. **i**–**l** Callose was not present at the apical tips of WT and si1*PhCRT* pollen tubes. **k** Increased callose deposition in the subapical zone of elongated si1*PhCRT* pollen tube (*double arrows* and *inset*). *Bars* 50 μm

About 2–2.5 h after pollen germination in the standard culture medium or the medium supplemented with si1*PhCRT*, cultivated pollen tubes appeared healthy and achieved comparable length. After this period, we observed the first signs of abnormal growth of si1*PhCRT* pollen tubes which intensified during the tube elongation. Elongated WT pollen tubes (cultivated about 4–5 h in standard medium) had a typical cylindrical shape with a clear zone in the growing tip (Fig. 1a). About 87% of them were straight and showed a typical bidirectional reverse-fountain cytoplasmic streaming pattern clearly visible under the light microscope (data not shown). In contrast, approximately 89% of si1*PhCRT*-treated pollen tubes exhibited disturbed morphology during their elongation (Fig. 1c–f) as well as reduced length and growth rate compared to WT tubes (online resources S1). Finally, more than 70% of extended si1*PhCRT* pollen tubes ruptured after about 4–5 h of cultivation (data not showed) and released their cytoplasm (Fig. 1f, arrow). We next performed detailed light microscopic analysis of si1*PhCRT*-treated pollen tubes and noted obvious structural abnormalities during their growth. Shorter si1*PhCRT* tubes (cultivated about 2–2.5 h) had usually swollen tips (Fig. 1c, arrow); most of them were bent and contained transparent elements (starch-refractile amyloplasts or small vacuoles) in the cytoplasm (Fig. 1d, arrows). Longer si1*PhCRT* pollen tubes exhibited the similar morphological defects, including widely swollen tips (Fig. 1e), twists in the shank (Fig. 1e–f, arrowheads), and highly vacuolated cytoplasm even in the distal shank (Fig. 1e). All these structural abnormalities were intensified during elongation and correlated with a progressive loss of the clear zone at the growing pollen tube tip (Fig. 1e, arrow). Thus, we identified this phenotype as the typical for elongating si1*PhCRT* pollen tubes. Just before rupture of the membrane/cell wall, the rapid bidirectional cytoplasmic streaming in the shank of extended si1*PhCRT* tubes stopped and the bulk cellular contents moved in a massive surge towards the apex (Fig. 1e, arrow). Control experiments revealed that neither 1.5 nM scr_si1*PhCRT* (Fig. 1g) nor ssDNA (Fig. 1h) had any impact on pollen tube morphology or elongation. It should be noted that pollen germination was blocked in the medium supplemented with si1*PhCRT*, but when the duplex was added to the culture medium containing outgrowing pollen tubes (Fig. 1b), the tubes were able to extend for about 4–5 h (Fig. 1e). We have also found that the highest tested concentration of si1*PhCRT* (18 nM) arrested the growth of pollen tubes before their length exceeded the diameter of the pollen grain. All these results suggest that mature *Petunia* pollen grains do not contain enough CRT protein to complete pollen tube elongation but must start translation of long-lived mRNAs just after pollen hydration. Moreover, we cannot exclude that si1*PhCRT* concentration of 18 nM may cause off-target effect.

To examine the effect of *PhCRT* knockdown on callose deposition, we used aniline blue to stain pollen tubes growing in different culture conditions. As expected, in most of the WT and scr_si1*PhCRT* pollen tubes (almost 90%, data not showed), callose was uniformly distributed along the entire tube shank except at the elongating tip (Fig. 1i, l, respectively). We observed a similar pattern of callose distribution in shorter si1*PhCRT*-treated tubes (Fig. 1j), but in elongated si1*PhCRT* pollen tubes (almost 70%, data not showed) we revealed increased callose deposition in the subapical region (Fig. 1k, *double arrows*, *inset*).

**Fig. 2:**
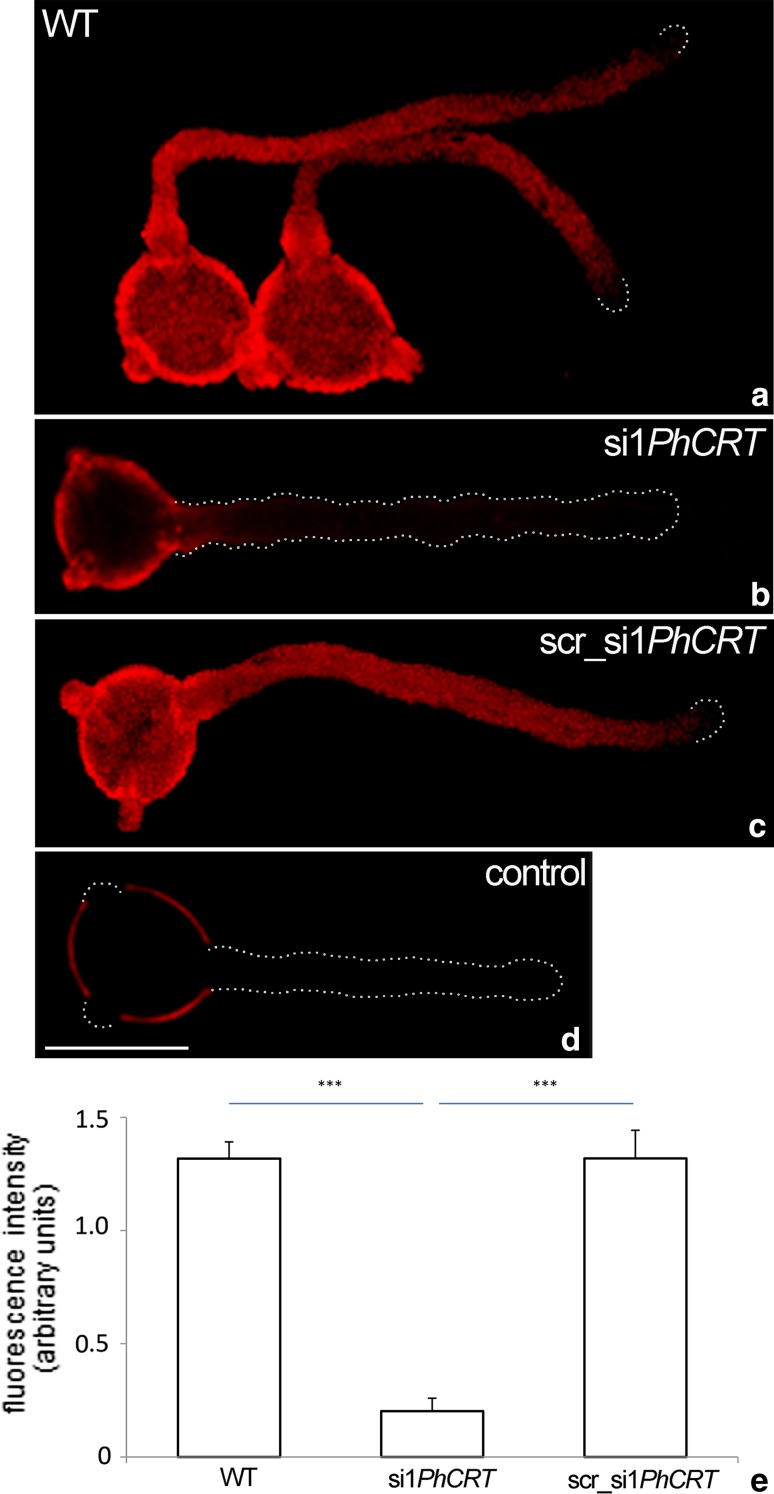
Quantitative analyses of *PhCRT* mRNA in *Petunia* WT (**a**), si1*PhCRT* (**b**), and scr_si1*PhCRT* (**c**) elongated pollen tubes. Both in WT and scr_si1*PhCRT* pollen tubes, *PhCRT* transcripts were diffusely distributed throughout the tube shank, extending from the base to the subapical zone (**a**, **c**). In contrast, si1*PhCRT* pollen tube was almost devoid of hybridization signals (**b**). **d** Negative FISH control. **e** The level of *PhCRT* transcripts was 85% lower in si1*PhCRT* pollen tubes compared to WT and scr_si1*PhCRT* tubes. Graphs show the relative *PhCRT* mRNA levels in pollen tubes elongating in different culture conditions (mean of 20 replicates for each experiment variant and standard deviation). Arbitral units on the *y*-axis show total FISH intensity (pixel). Statistical analysis was carried out by one-way ANOVA (***P ≤ 0.001). *Bar* 50 μm

**Fig. 3:**
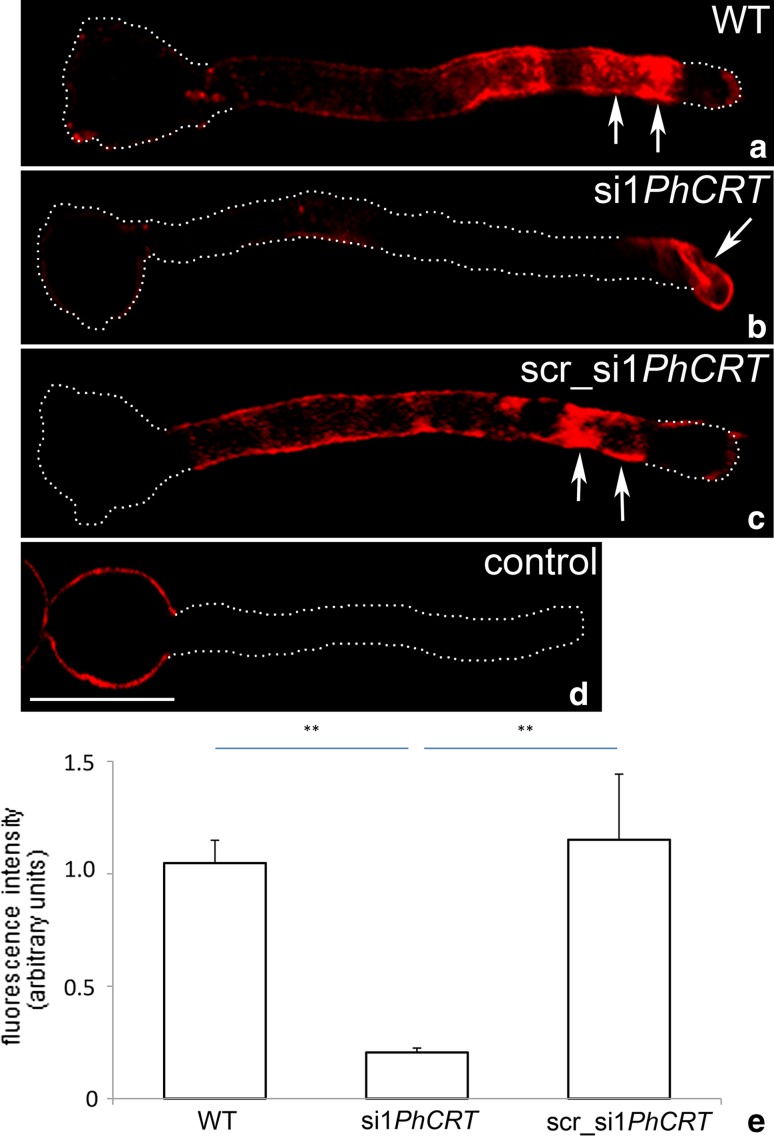
Quantitative analyses of CRT in *Petunia* WT (**a**), si1*PhCRT* (**b**), and scr_si1*PhCRT* (**c**) elongated pollen tubes. Both in WT and scr_si1*PhCRT* pollen tubes, CRT was preferentially localized in the distal regions, where intense labeling was observed adjacent to the peripheral cytoplasm of the tube and accumulated at the subapical domain (**a**, **c**, *arrows*), whereas the growing tips were lack of the labeling. In contrast, only residual immunofluorescence abnormally localized to the apical cytoplasm was detected in si1*PhCRT* pollen tube (**b**, *arrow*). **d** Negative immunocytochemistry control. **e** The level of CRT was 80% lower in si1*PhCRT* pollen tubes compared to WT and scr_si1*PhCRT* tubes. Graphs show the relative CRT levels in pollen tubes elongating in different culture conditions (mean of 20 replicates for each experiment variant and standard deviation). Arbitral units on the *y*-axis show total immunofluorescence intensity (pixel). Statistical analysis was carried out by one-way ANOVA (**P ≤ 0.01). *Bar* 50 μm

To confirm that the morphological defects in growing pollen tubes were the result of post-transcriptional *PhCRT* silencing, we first performed FISH to detect *PhCRT* mRNA in elongated pollen tubes 4–5 h after addition of si*1PhCRT* or scr_si1*PhCRT*. In WT and scr_si1*PhCRT* tubes, these transcripts were localized in the pollen grain cytoplasm and were diffusely distributed throughout the tube shank, extending from the base to the subapical zone (Fig. 2a, c, respectively). In contrast, si1*PhCRT* pollen tubes were nearly devoid of hybridization signals (Fig. 2b). As shown in Fig. 2e, the level of *PhCRT* transcripts was about 85% lower in elongated si1*PhCRT* pollen tubes than in WT and scr_si1*PhCRT* tubes. Next, we performed immunofluorescence staining to examine the effects on CRT protein level in cultivated pollen tubes. In both WT and scr_si1*PhCRT* pollen tubes, CRT was preferentially localized in the distal regions (Fig. 3a, c, respectively), where intense labeling was observed adjacent to the peripheral cytoplasm of the tube and accumulated at the subapical zone (Fig. 3a, c, *arrows*). In contrast, only residual immunofluorescence abnormally localized to the apical cytoplasm was detected in elongated si1*PhCRT* pollen tubes (Fig. 3b, *arrow*). Quantitation of the immunofluorescence signal revealed comparable levels of CRT in WT and scr_si1*PhCRT* pollen tubes, but the level of CRT was reduced up to 80% in si1*PhCRT* pollen tubes (Fig. 3e). Thus, our microscopic quantitative analyses demonstrated that *Petunia* pollen tubes efficiently took up si1*PhCRT* from the culture medium, resulting in dramatic reductions of *PhCRT* transcripts and CRT protein.

**Fig. 4:**
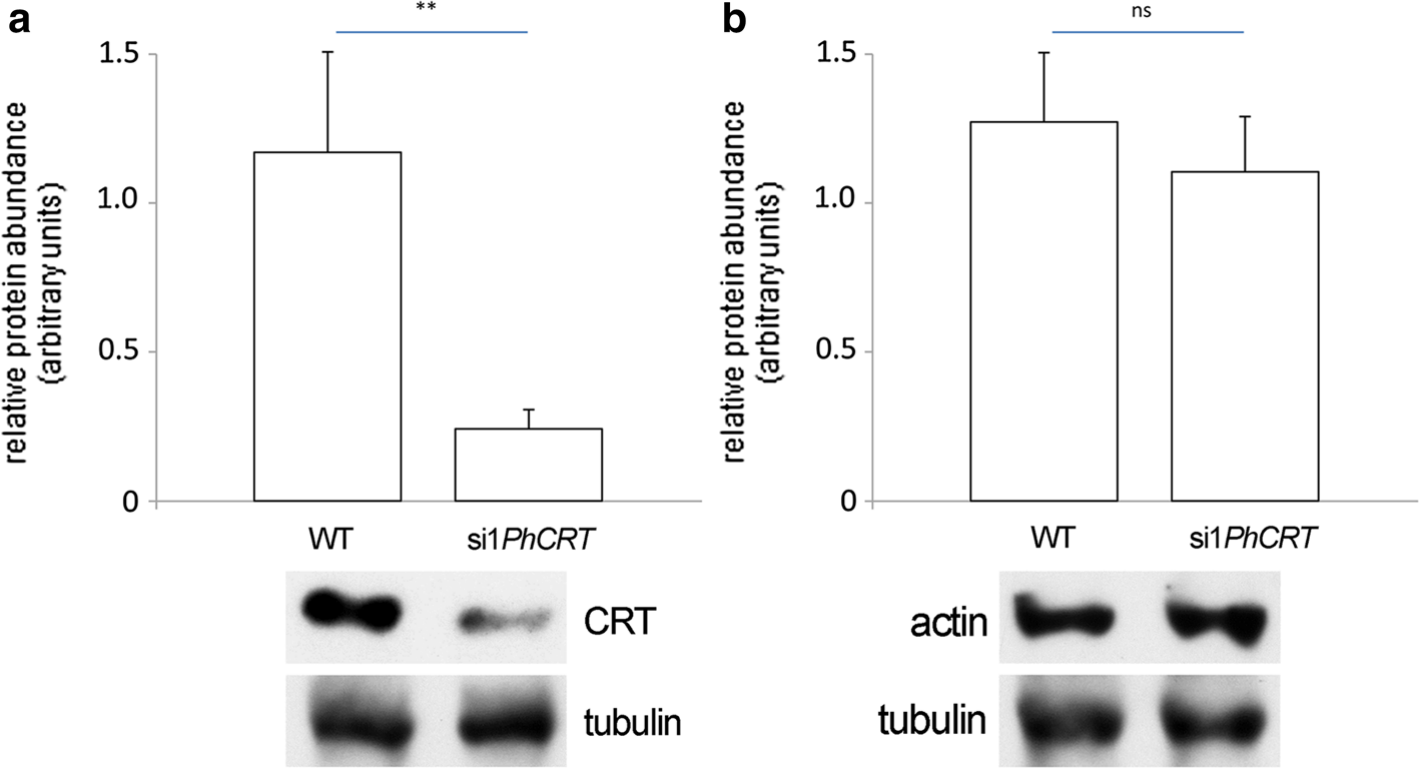
Western-blot analysis of CRT (**a**) and actin (**b**) levels in *Petunia* elongated WT and si1*PhCRT* pollen tubes. **a** The level of CRT was 79% lower in si1*PhCRT* pollen tubes compared to WT tubes. **b** The level of actin remained constant in WT and si1*PhCRT* pollen tubes. *Graphs* show the relative CRT and actin levels in pollen tubes elongating in different culture conditions (mean of five replicates and standard deviation) normalized to the *Petunia* tubulin levels. Statistical analysis was carried out by the Mann–Whitney tests (**P ≤ 0.01; *ns* not significant). Representative western blots are shown under the graphs

To confirm that si1*PhCRT* selectively degraded *PhCRT* mRNA and did not affect the expression of other proteins, we extracted total proteins from in vitro elongated WT and si1*PhCRT* pollen tubes and performed immunoblotting experiments with CRT PAb, anti-actin, and anti-tubulin antibodies. As shown in Fig. 4, Western blot analysis confirmed that CRT expression was decreased up to 79% in si1*PhCRT* pollen tubes compared to WT tubes (Fig. 4a). By contrast, the levels of actin and tubulin (reference proteins that are both required for proper pollen tube growth) remained constant in WT and si1*PhCRT* elongated tubes (Fig. 4b). Together, these results indicate that morphological abnormalities observed during si1*PhCRT* pollen tubes elongation are the result of PTGS, and that CRT is required for normal pollen tube growth in vitro.

### Knockdown of *PhCRT* causes several ultrastructural defects in elongating pollen tubes

Given our observation that cytoplasmic streaming stopped in si1*PhCRT* pollen tubes before they ruptured and the fact that bidirectional cytoplasmic streaming is required for cytoplasmic zonation of growing pollen tubes, we reasoned that si1*PhCRT* tubes likely had ultrastructural defects. To explore this possibility, we performed high-resolution electron microscopy on ultrathin longitudinal sections of WT and si1*PhCRT* elongated *Petunia* pollen tubes.

**Fig. 5:**
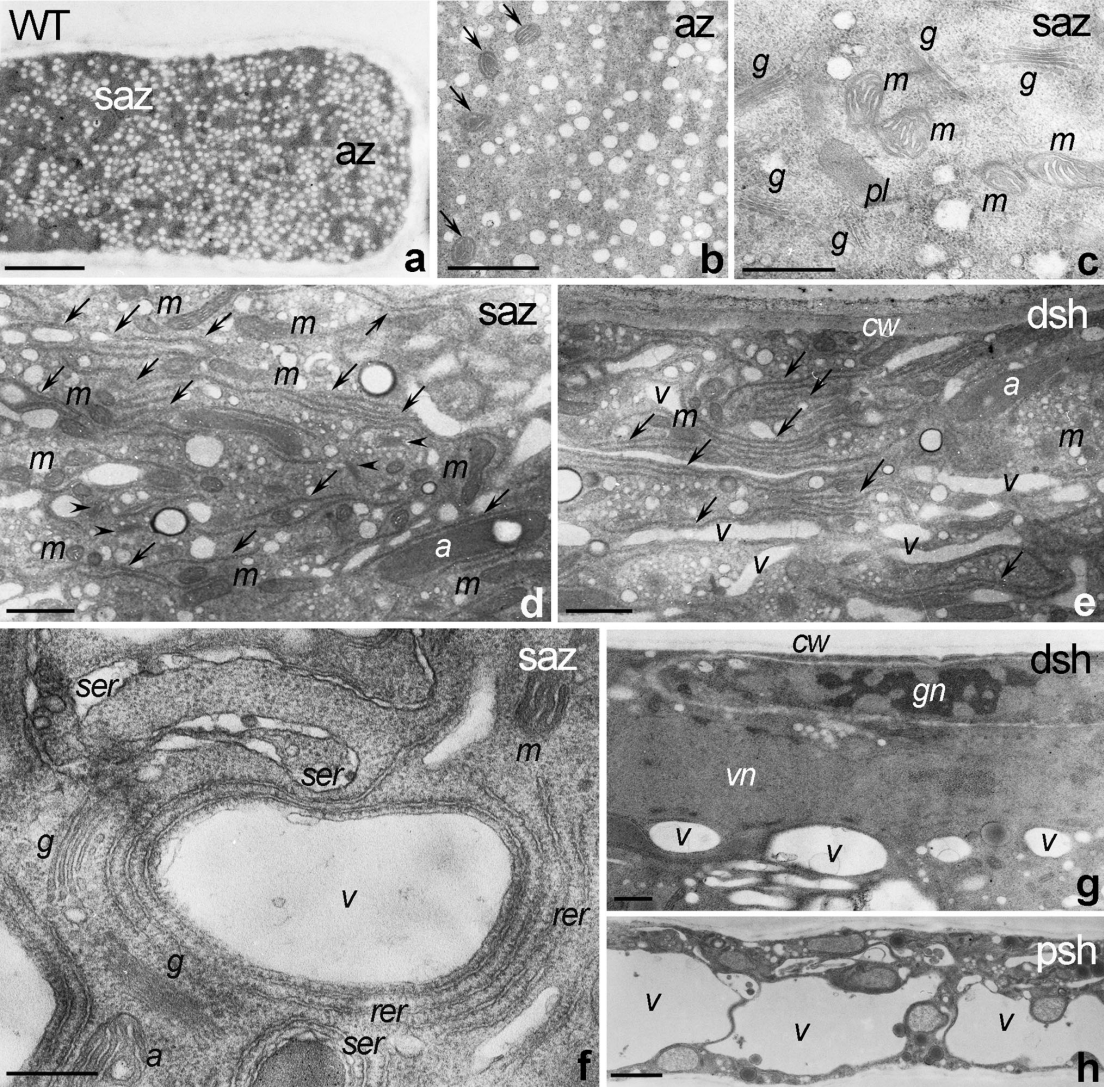
Ultrastructure of *Petunia* WT pollen tubes. **a**, **b** The clear apical zone (*az*) is packed with vesicles and occasionally contains small organelles such as mitochondria (*arrows* in **b**). **c**, **d**, **f** The subapical zone (*saz*) contains many metabolically active organelles such as mitochondria (*m*), Golgi stacks (*g* or *arrowheads* in **d**), and well-developed ER (*arrows* in **d**), including rough rER (*rer*) and smooth sER (*ser*). **e**, **g** Well-developed ER structures (*arrows* in **e**) are also present in the distal shank (*dsh*) where MGU composed of the vegetative and generative nuclei (*vn* or *gn*, respectively) is localized (**g**). **h** The proximal shank (*psh*) is highly vacuolated. *a* amyloplast, *cw* cell wall, *v* vacuoles. *Bars* 2 μm (**a**, **h**), 1 μm (**b**, **d**, **e**, **g**), 500 nm (**c**), 200 nm (**f**)

WT pollen tubes showed the expected ultrastructural features. The apical zone was clear and packed with vesicles (Fig. 5a, b), and occasionally contained small organelles such as mitochondria (Fig. 5b, arrows). In the subapical zone, we observed many metabolically active organelles, including mitochondria (Fig. 5c, d), Golgi stacks (Fig. 5c, d, arrowheads), and well-developed ER structures (Fig. 5d, arrows). Additionally, granular ribosome-rich rER and tubular sER were abundant in the subapical zone (Fig. 5f). The distal shank contained extended ER cisternae (Fig. 5e, arrows), numerous small vacuoles (Fig. 5e, g), and the MGU containing the vegetative nucleus and the generative cell (Fig. 5g). Finally, the proximal shank of elongated WT pollen tubes was full of large vacuoles (Fig. 5h).

**Fig. 6:**
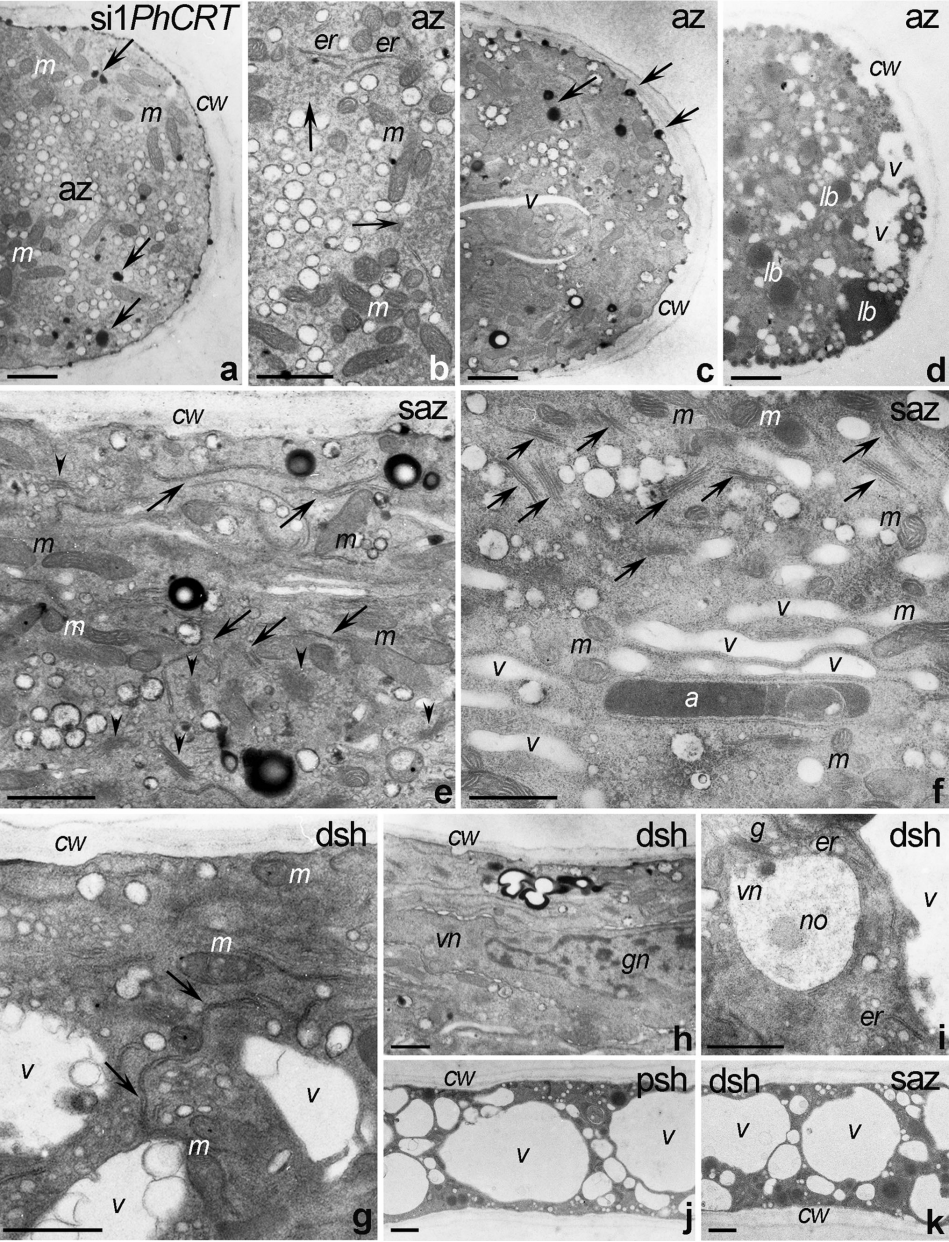
Ultrastructure of *Petunia* si1*PhCRT* pollen tubes. **a**–**d** The apical zone (*az*) contains numerous mitochondria (*m*), very short or even fragmentized/disorganized ER (*er* and *arrows* in **b**), electron-dense vesicles (*arrows* in **a**, **c**), vacuoles (*v*), and lipid bodies (*lb*). **e**, **f** In the subapical zone (*saz*), only single ER cisternae (*arrows* in **e**) are present; mitochondria (*m*) and dictyosomes (*arrowheads* in **e**) are normally localized in *saz*; however, dictyosomes are observed mainly in the peripheral cytoplasm (*arrows* in **f**). **g**-**i** The distal shank (*dsh*) is highly vacuolated and occasionally contains ER (*arrows* in **g** and *er* in **i**), two-phase vesicles with electron-dense cortices (*arrow* in **h**), and normally positioned MGU (**h**) composed of the vegetative and generative nuclei (*vn* or *gn*). **j** The proximal shank (*psh*) is highly vacuolated. *a* amyloplast, *cw* cell wall. *Bars* 1 μm

Numerous aspects of pollen tube apical domain ultrastructure were affected by knockdown of *PhCRT*. This zone contained numerous mitochondria (Fig. 6a, b) and ER (Fig. 6b). However, the ER cisternae at the tip were very short, indicating that they were highly fragmentized/disorganized (Fig. 6b, arrows). We did not observe any obvious ultrastructural changes in the mitochondria except that they tended to aggregate (Fig. 6b). Additionally, numerous electron-dense vesicles were visible in the apical domain of si1*PhCRT*-treated pollen tubes (Fig. 6a, c, arrows). As si1*PhCRT* pollen tubes grew, the number of electron-transparent vesicles decreased in the clear zone (Fig. 6c), suggesting that vesicle trafficking was disrupted, but small vacuoles appeared in the tip cytoplasm (Fig. 6c, d). Finally, we observed many lipid bodies in the apex of elongating si1*PhCRT* pollen tubes (Fig. 6d). We also detected several defects in the subapical zone and distal shank of abnormally growing si1*PhCRT* pollen tubes (Fig. 6e–i). In elongated si1*PhCRT*-treated tubes, ER cisternae were substantially shorter than in WT (Fig. 6e, arrows) and we found many two-phase vesicles with electron-dense cortices localized in the cytoplasm (Fig. 6e). Mitochondria and Golgi stacks (Fig. 6e, arrowheads) were localized normally in the subapical cytoplasm of these tubes. However, we observed many dictyosomes (Fig. 6f, arrowheads) and elongated small vacuoles (or distended ER cisternae) accumulated in the peripheral cytoplasm of the sub-apex. The distal shank of elongated si1*PhCRT* pollen tubes was highly vacuolated and contained a few reduced ER structures (Fig. 6g, arrows). However, the MGU was positioned normally in this domain (Fig. 6h), and the nucleolus was clearly visible within the vegetative nucleus (Fig. 6i). At the final growth stage, usually just before si1*PhCRT* pollen tube rupture, the subapical domain and both the proximal and distal shank zones were highly vacuolated (Fig. 6j, k). Together, our data indicate that CRT is required in growing pollen tubes for vesicle trafficking, organelle transport/positioning, and proper ER ultrastructure.

### CRT is required for normal actin cytoskeleton structure in elongating pollen tubes

**Fig. 7:**
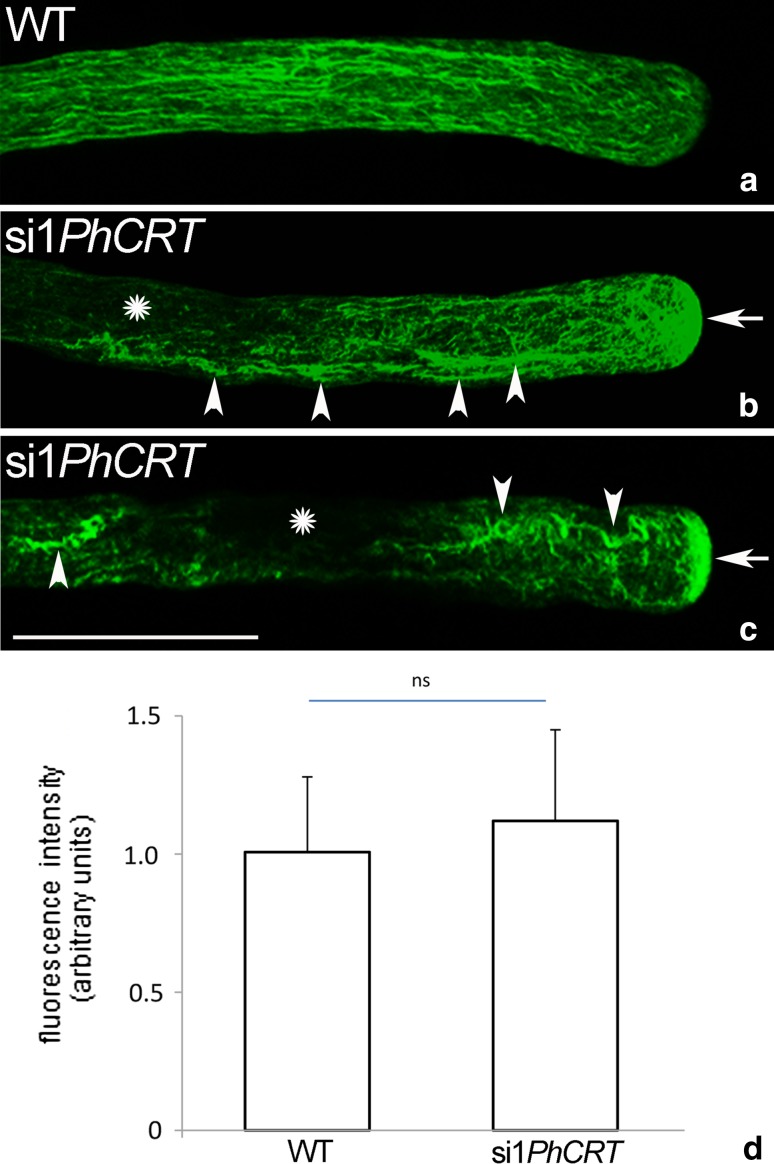
Quantitative analyses of F-actin in *Petunia* WT (**a**) and si1*PhCRT* (**b**, **c**) elongated pollen tubes. In WT pollen tubes, long AFs were distributed throughout the entire tube in a net axial array that was largely parallel to the direction of elongation, except at the very tip (**a**). By contrast, long actin bundles disappeared in the shank of si1*PhCRT*-treated pollen tubes (*arrowheads* in **b** and **c**), but abnormal actin aggregates are observed in the apical cytoplasm (*arrows* in **b** and **c**). Additionally, some “empty spaces” (probably vacuoles) are present in the distal shank of si1*PhCRT* pollen tubes (*stars* in **b** and **c**). Quantitation of staining intensity revealed that total F-actin amount was not affected by knockdown of *PhCRT* expression (**d**). *Graphs* show the relative F-actin levels in pollen tubes elongating in different culture conditions (mean of 20 replicates for each experiment variant and standard deviation). Arbitral units on the *y*-axis show total immunofluorescence intensity (pixel). Statistical analysis was carried out by the Mann–Whitney tests (*ns* not significant). *Bar* 25 μm

**Fig. 8:**
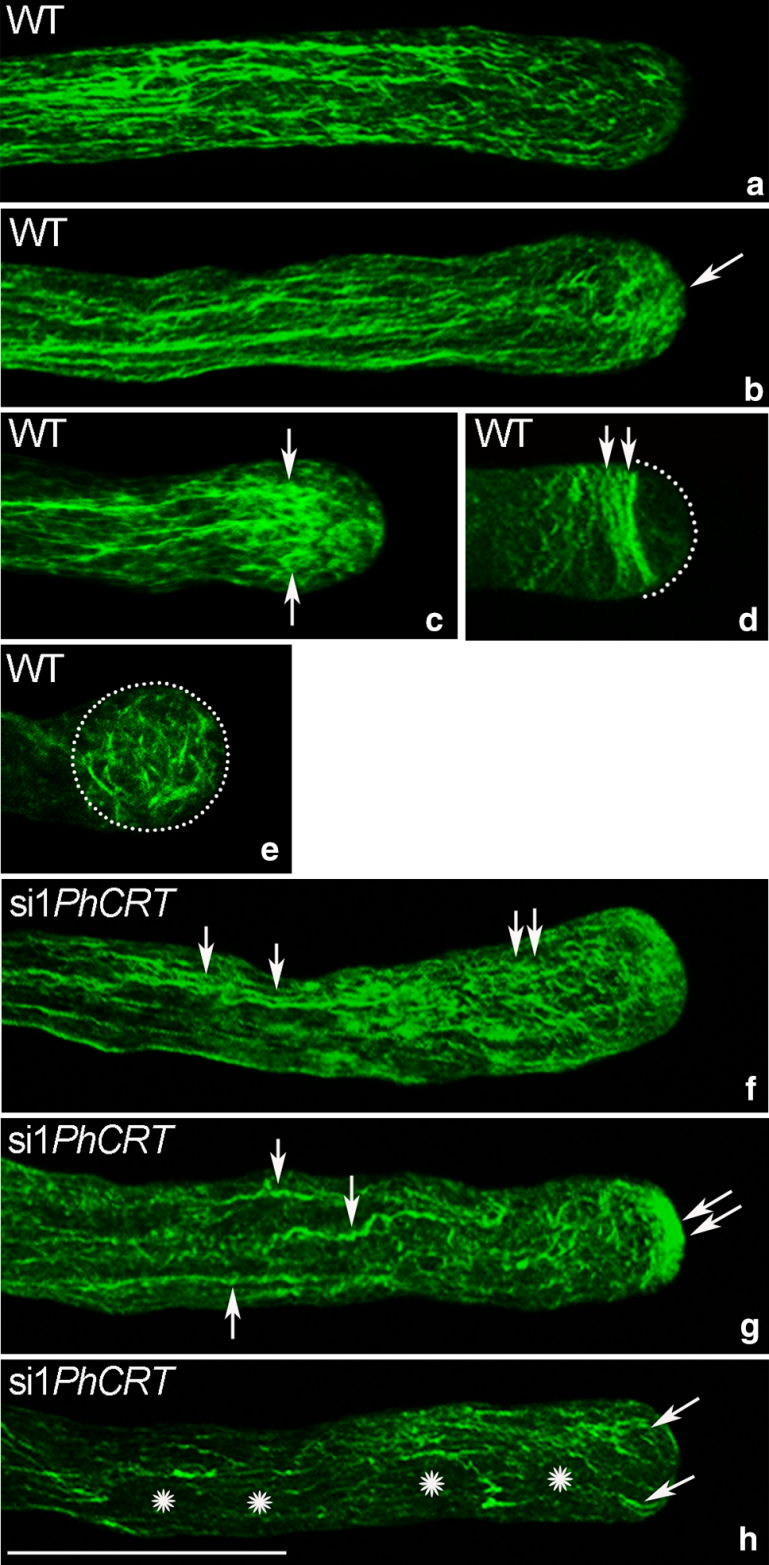
F-actin staining in *Petunia* WT (**a**–**e**) and si1*PhCRT* (**f**–**h**) elongated pollen tubes. **b**, **e** Dynamic actin meshwork in WT pollen tube apical cytoplasm (*arrow* in **b** and *dotted circle* in **e**). **c**, **d** Distinct cortical fringe of densely packed AFs in the subapical zone of WT pollen tube (*arrows* in **c** and *double arrow* in **d**). **f**, **g** AFs in the shank of si1*PhCRT* pollen tubes are much shorter, thicker and twister (*arrows*) than in WT tubes (compare with **a** and **b**), and some of them are extended to the swollen apex. **g**, **h** Dense actin aggregates (*double arrow* in **g**) and single curved AFs (*arrows* in **h**) are present in the apical domain of si1*PhCRT* pollen tubes. Some dark spaces (probably vacuoles) appeared in the cytoplasm of si1*PhCRT* pollen tubes (*stars* in **h**). *Bar* 25 μm

Because knockdown of *PhCRT* affected cytoplasmic streaming, proper organelle positioning, and vesicle transport, which are actin-dependent processes, we wanted to examine the effect of loss of CRT on arrangement of AFs during pollen tube growth. First, we performed a comparative analysis of F-actin staining (visualized by AlexaFluor^®^488 Phalloidin) in pollen tubes elongated in different culture conditions. Our fluorescence staining of WT pollen tubes confirmed that long AFs were distributed throughout the entire tube in a net axial array that was largely parallel to the direction of elongation, except at the very tip (Fig. 7a). By contrast, long actin bundles were greatly reduced or almost absent from the shank of si1*PhCRT*-treated tubes (Fig. 7b, c, *arrowheads*), and we observed abnormal actin aggregates in their apical cytoplasm (Fig. 7b, c, *arrows*). Moreover, we often observed “empty spaces” (probably large vacuoles) in the distal shank of elongated si1*PhCRT* tubes (Fig. 7b, c, *stars*). Despite these alterations in AF organization, quantitation of staining intensity revealed that total F-actin amount was not affected by knockdown of *PhCRT* expression (Fig. 7d). At higher magnification, we could more clearly discern the effect of loss of CRT on actin organization. In the shank of WT pollen tubes, AFs were typically organized in long actin bundles parallel to the long axis, both in the center and at the periphery of the tube (Fig. 8a, b). As expected from previous reports, these actin bundles did not extend into the apical clear zone. Instead, we often observed much shorter AFs (Fig. 8b, arrow) and a fine actin meshwork (Fig. 8e, dotted circle) in the tube apical cytoplasm and a distinct cortical fringe of densely packed AFs in the subapical zone (Fig. 8c, d, arrows or double arrow, respectively). In the shank of si1*PhCRT* pollen tubes, AFs were much shorter and thicker (Fig. 8f, g, arrows) than in WT tubes; these AFs lost their specific configuration and were almost completely absent in highly elongated si1*PhCRT* pollen tubes (Fig. 8h). In the presence of si1*PhCRT* in the culture medium, the prominent actin fringe (normally existing behind the tube tip) was absent. By contrast, some shorter AFs extended to the swollen tip (Fig. 8f, double arrow); they were present centrally and peripherally in the subapical domain and disappeared during tube elongation (Fig. 8g, h). Instead of a fine meshwork of F-actin in the apical domain, we observed dense actin aggregates in si1*PhCRT* pollen tubes (Fig. 8g, double arrow). As the si1*PhCRT*-treated pollen tubes elongated, these F-actin structures also disappeared; only single curved AFs were present in the apical cytoplasm (Fig. 8h, arrows), and some dark spaces (probably vacuoles) appeared in the cytoplasm of elongated si1*PhCRT* pollen tubes (Fig. 8h, stars). Together, these results indicate that loss of CRT does not affect the expression level of actin, but CRT is strictly required for proper actin cytoskeleton organization in defined pollen tube zones.

### CRT is critical for stabilization of the tip-focused Ca^2+^ gradient in growing pollen tubes

**Fig. 9:**
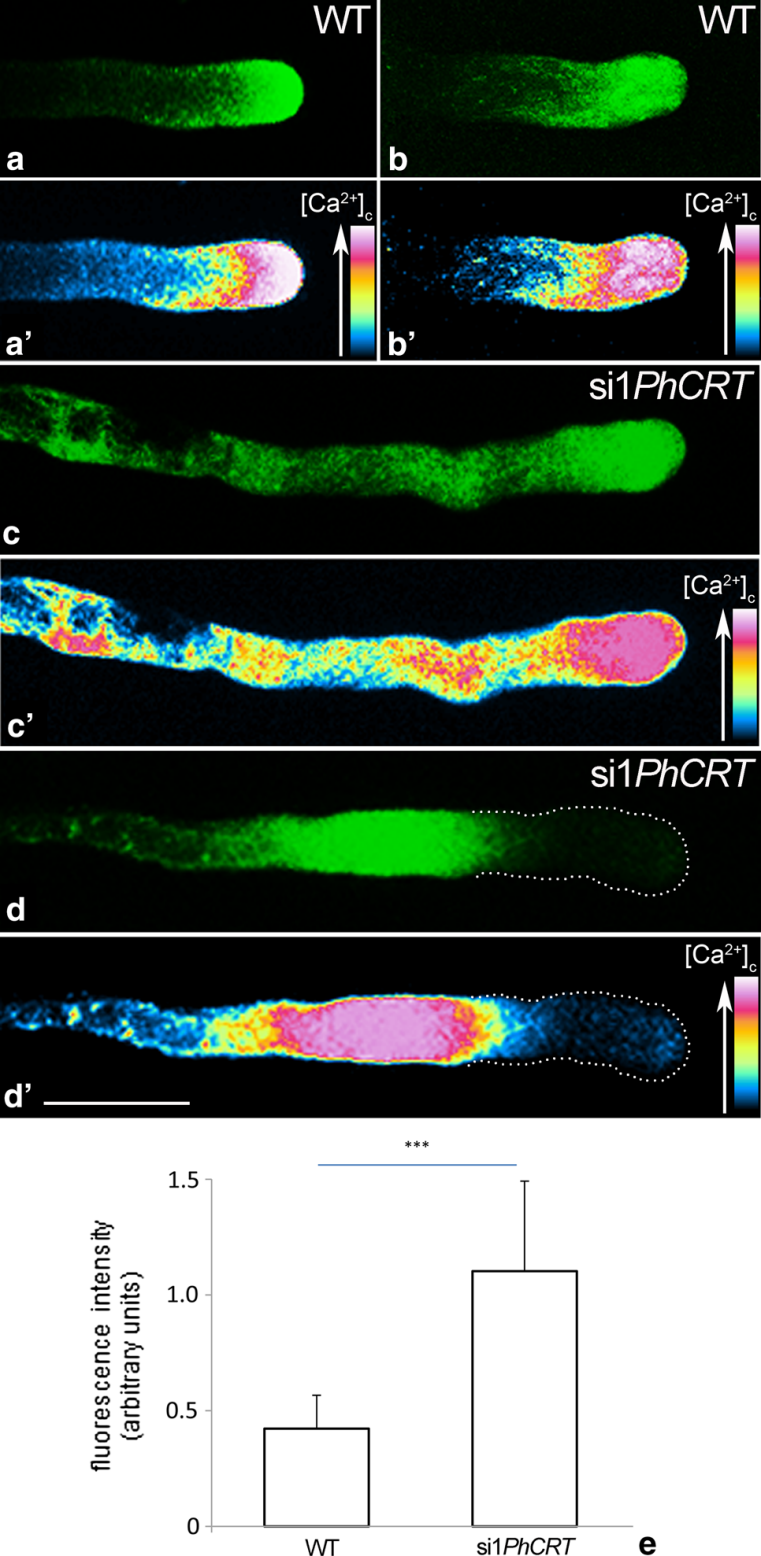
Intracellular Ca^2+^ detection in WT and si1*PhCRT* elongating pollen tubes using the fluorescent Ca^2+^ indicator fluo-4/AM. Control pollen tubes exhibit the typical tip-focused Ca^2+^ gradient (**a**–**b**′), while steep cytosolic Ca^2+^ gradient was disrupted in pollen tubes treated with si1*PhCRT* (**c**–**d**′). **e** A significant increase in the level of fluorescence intensity was observed in whole si1*PhCRT* pollen tubes compared to control tubes. *Graphs* show the relative Ca^2+^ fluorescence in pollen tubes elongating in different culture conditions (mean of 20 replicates for each experiment variant and standard deviation). Arbitral units on the *y*-axis show total fluorescence intensity (pixel). Statistical analysis was carried out by the Mann–Whitney tests (***P ≤ 0.001). *Bar* 25 μm

Because the cytosolic Ca^2+^ gradient is necessary for structural organization of the actin cytoskeleton in angiosperm pollen tubes, we loaded in vitro elongating pollen tubes with fluo-4/AM to test the possible effects of si1*PhCRT* treatment on the Ca^2+^ distribution. As expected pollen tubes cultured in the standard medium exhibited a tip-focused Ca^2+^ (Fig. 9a, b′), but this steep Ca^2+^ gradient disappeared in elongating si1*PhCRT* pollen tubes (Fig. 9c, d′). In these tubes, we commonly observed two abnormal patterns of Ca^2+^ distribution. Some of the si1*PhCRT* pollen tubes showed stronger fluorescence within the whole shank and swollen tips (Fig. 9c, c′), while other tubes treated with si1*PhCRT* showed very weak fluorescence at their tips and abnormal Ca^2+^ accumulation in the subapical zone and distal shank (Fig. 9d, d′). Moreover, we observed a significant higher level of the fluorescence intensity in whole si1*PhCRT* pollen tubes than in control tubes (Fig. 9e). These results clearly showed that loss of CRT led to the disappearance of the tip-focused Ca^2+^ gradient and, consequently, disruption of proper AF organization in defined pollen tube zones.

## Discussion

Previous work demonstrating that CRT is expressed in germinating pollen and elongating tubes both in vivo and in vitro suggested that plant CRT plays an important role in pollen tube growth (Lenartowska et al. 2002, 2009; Nardi et al. 2006; Lenartowski et al. 2014, 2015; Suwińska et al. 2015). Two main functions have been suggested for CRT in this process: (1) CRT’s Ca^2+^-binding/buffering properties may contribute to regulation of Ca^2+^ homeostasis, which is critical for pollen tube tip growth, and (2) CRT’s chaperone activity may facilitate the high rate of protein synthesis required for extremely fast elongation of the tube. Our present results provide support for both of these proposed functions of CRT.

### Exogenous delivery of *PhCRT*-specific siRNA is effective and impairs pollen tube elongation

PTGS is an evolutionarily conserved and homology-dependent RNA degradation mechanism in which short RNA sequences (e.g., siRNAs) are used for defense against pathogens, regulation of gene expression, and silencing of repeats. Proteome and transcriptome analyses have confirmed that the PTGS pathway components exist in many plant cell types, including pollen and pollen tubes. Moreover, experiments with transgenic plants expressing siRNA constructs further indicate that PTGS pathway components are functional in pollen tubes (see reviews by Le Trionnaire et al. 2011; He et al. 2015). The following reasons led us to think that in vitro cultivated *Petunia* pollen tubes could take up specific siRNAs directly from the culture medium. First, endocytic pathways are active in the growing domain of the tube and are required for proper pollen tube elongation (see review by Onelli and Moscatelli 2013). Second, Tenllado et al. (2003) applied a bacterial crude extract containing virus-derived double-stranded RNAs (dsRNAs) to *Nicotiana* leaves and showed that the dsRNAs promoted specific interference and prevented further infection in tobacco. Third, both local and systemic PTGS in plants occurred more quickly and extensively upon targeting the apical meristem than mature leaves (Dalakouras et al. 2016). This observation is not surprising because the apical meristem cells have a very thin, flexible, and extensible primary cell wall that is freely permeable to small molecules, including small proteins. This cell wall structure is similar to that at the apex of growing pollen tubes. Fourth, plant cells, including root hairs and growing pollen tubes can take up DNA oligonucleotides (Paungfoo-Lonhienne et al. 2010). Fifth, our previous work showed that cell wall digestion was not required for successful angiosperm pollen tubes to take up myosin II subfragment 1 from culture medium (Lenartowska and Michalska 2008). Thus, we could label AFs in pollen tubes as well as could be done in tip-growing root hairs (Tominaga et al. 2000). Finally, Khatri and Rajam (2007) demonstrated that the tip-growing germ tubes of fungal spores can take up siRNA directly from the culture medium. It was also confirmed for water fern *Marsilea* microspores which were able to get dsRNA from the medium at the time of their hydration (Klink and Wolniak 2001; Boothby et al. 2013). Together, these results indicate that siRNA can overcome the barrier of the primary cell wall to enter the cytoplasm.

Here, our quantitative analyses clearly showed that *Petunia* pollen tubes in vitro took up si1*PhCRT*, resulting in degradation of *PhCRT* transcripts (Fig. 2). Consequently, the level of CRT protein in si1*PhCRT* elongated tubes decreased dramatically, as evidenced by greatly reduced levels of CRT–CRT PAb complexes in elongating *Petunia* pollen tubes (Fig. 3). This efficient CRT PTGS caused severe morphological and ultrastructural abnormalities, including disorganization of the actin cytoskeleton (Fig. 8). In contrast, si1*PhCRT* did not affect the levels of actin and tubulin (Fig. 4), confirming that si1*PhCRT* selectively knocked down *PhCRT*. Moreover, si1*PhCRT* did not block global transcription in elongating pollen tubes, as evidenced by the presence of nucleoli in their vegetative nuclei. Although si1*PhCRT* pollen tubes eventually ruptured, we do not interpret this as a result of stress for two reasons. First, the tubes continued growing for up to 4–5 h. Second, we did not find deposition of callose at the tube tips (Fig. 1), a well-characterized response to wounding or toxic conditions (Bhuja et al. 2004; Speranza et al. 2009; Wang et al. 2009b; Sheng et al. 2012; Fang et al. 2016a, b), and a common indicator of incompatible pollen (Guyon et al. 2004). It has been suggested that callose deposition at the pollen tube tip is induced by a sudden increase of [Ca^2+^]_c_ in the apical cytoplasm (Bhuja et al. 2004). In contrast, si1*PhCRT* pollen tubes exhibited increased accumulation of callose at the subapical zone, not at the growing tip. Given that CRT is known to play a key role in regulating Ca^2+^ homeostasis in eukaryotic cells (see reviews by Jia et al. 2009; Michalak et al. 2009), lack of this Ca^2+^-binding/buffering protein in si1*PhCRT* pollen tubes may result in local elevated [Ca^2+^]_c_. In fact, we confirmed that si1*PhCRT* elongating tubes lose the tip-focused Ca^2+^ gradient (Fig. 9). Thus, we argue that in the absence of CRT, which normally accumulates in the abundant ER and Golgi compartments in the sub-apex (Suwińska et al. 2015), excess Ca^2+^ in the subapical zone leads to the accumulation of callose in this region.

### CRT is required for polarized pollen tube cytoplasm and proper actin cytoskeleton structure

Pollen tubes cultured in standard germination medium usually exhibit cylindrical, uniaxial structures with highly polarized cytoplasmic organization including a vesicle-packed clear apical zone in which vacuoles are excluded and an organelle-rich subapical domain (see review by Qin and Yang 2011). Here, we show that knockdown of *PhCRT* prevented pollen tube growth (online resources S1) and strongly affected tube morphology (Fig. 1) and ultrastructure (Fig. 6). We observed twisted si1*PhCRT* pollen tubes in which the diameters were irregular and wide, the tips were expanded, and cytoplasmic streaming arrested just before rupture. One of the most obvious ultrastructural phenotypes was that numerous mitochondria, ER, lipid bodies, and even vacuoles penetrated the bulging apex, leading to a loss of the clear zone normally found at the pollen tube tip.

It has been long recognized that a prime candidate for regulation of pollen tube polar growth is the actin cytoskeleton; at a minimum, it controls cytoplasmic streaming/organelle positioning and appears to be involved in apical docking of the Golgi-derived vesicles (see reviews by Fu 2010; Hepler et al. 2013; Cai et al. 2015; Qu et al. 2015). Excellent experiments using the F-actin assembly inhibitor latrunculin-B and different organelle markers confirmed that movement of mitochondria, ER, and vacuoles is dependent on the actin cytoskeleton in lily pollen tubes (Lovy-Wheeler et al. 2007). These authors also showed that the ER and mitochondria move apically in the cortical region of the tube along longitudinal actin cables and the actin fringe. These organelles become enriched in the subapical domain just behind the clear zone where growth occurs, and some of them reverse direction and move basipetally through the central core of the tube. In contrast, vacuoles are located further away from the growing tip and never extend to the growing domain. Studies on pollen tubes from different species have also confirmed that the fast movement of mitochondria and Golgi-derived vesicles is actin dependent (Romagnoli et al. 2007) and that mitochondria, ER, dictyosomes, and vesicles use myosins to drive their motions (Holweg and Nick 2004; Lovy-Wheeler et al. 2007; Romagnoli et al. 2007; Zheng et al. 2010). Furthermore, the cortical actin fringe acts as a track along which myosin can transport vesicles to the tube apex, thus contributing to polarized growth (Cárdenas et al. 2008). All these results indicate that the acto-myosin system is required for organelle/vesicle trafficking throughout the tube, and that the subapically located actin fringe plays a crucial role in their selective distribution to the growing domain. On the one hand, this structure delivers secretory vesicles to the clear zone, but, on the other hand, it stops most of the metabolically active organelles in the sub-apex and eliminates vacuoles from the tip. Our present work shows that lack of CRT causes dysfunction of intracellular transport. This work also provides clear evidence that *PhCRT* knockdown causes disorganization of F-actin structures in all distinct cytoplasmic zones of *Petunia* pollen tubes (Fig. 8). We argue that this F-actin disruption impairs intracellular transport and prevents proper pollen tube elongation. Additionally, our work lends support to speculations of Nardi et al. (2006) about the probable role of CRT in cytoplasmic streaming in *Nicotiana* pollen tubes.

We also found that instead of containing fine AFs, the apical domain of si1*PhCRT* pollen tubes contained short, disorganized actin fragments that tended to aggregate. This apical F-actin accumulation was accompanied by substantially fewer vesicles and abnormal presence of residual CRT in the clear zone of shorter pollen tubes. This actin aggregates were not present in fully elongated si1*PhCRT* tubes. Normally, secretory vesicles containing cell wall precursors are transported to the growing domain of the tube by highly dynamic AFs and myosin XI (Lovy-Wheeler et al. 2007; Cárdenas et al. 2008). Thus, we conclude that the knockdown of *PhCRT* expression resulted in dysfunction of exocytosis and/or vesicle trafficking/docking and, consequently, arrested tube elongation. Consistent with this idea, pollen tube growth is inhibited whenever AFs, particularly the tip-localized F-actin array, are disorganized (Vidali et al. 2001; Cárdenas et al. 2005, 2008; Chen et al. 2007). Pollen tube tip growth is thought to be dependent on a balance between turgor pressure and extensibility of the primary cell wall (Kroeger et al. 2011). Generally, fusion of vesicles with the plasma membrane allows the plant cell wall to expand (see review by Geitmann and Ortega 2009). If the cortical actin fringe and the apical actin meshwork in the growing domain of the pollen tube function together to deliver secretory vesicles to specific sites on the apical dome, this targeted secretion could locally weaken the cell wall and allow turgor-driven tip expansion. However, when apical AFs are destroyed, vesicles are no longer precisely targeted and do not modify the local cell wall. Alternatively, impaired secretion may cause reduction of cell wall extensibility by increasing its thickness. In fact, we observed several abnormalities, such as dysfunction of shank-localized and tip-localized AFs, incorrect organelle positioning, and substantially fewer vesicles in the clear zone in elongating si1*PhCRT* pollen tubes. The eventual rupturing of the cell wall may have occurred as a result of imbalance between cytoplasmic streaming, turgor pressure, and extensibility of the cell wall.

We propose that the mechanism underlying CRT’s function in pollen tube growth involves ABPs, which are required for the formation and maintenance of both the long actin cables in the shank and actin dynamics in the tube apex. Most of the ABPs are regulated by Ca^2+^ (see reviews by Ren and Xiang 2007; Fu 2010; Staiger et al. 2010; Hepler and Winship 2015; Qu et al. 2015). For example, the villin/gelsolin proteins cross link and stabilize actin bundles in low [Ca^2+^]_c_ in the shank and subapical domain but actively fragment and promote F-actin depolymerization in high [Ca^2+^]_c_ in the pollen tube tip. Profilin binds G-actin and regulates F-actin polymerization. However, in pollen tube regions of high [Ca^2+^]_c_, this ABP sequesters G-actin and prevents its polymerization into F-actin. Thus, in the tip, where [Ca^2+^]_c_ is high, villin/gelsolin and profilin would degrade the existing F-actin and prevent polymerization of new AFs. Additionally, elevated [Ca^2+^]_c_ can inhibit myosin XI ATPase activity and thus impair motility of organelles and vesicles (Tominaga et al. 2012). We previously reported that the sub-apex of the *Petunia* pollen tube, where [Ca^2+^]_c_ is much lower than at the tip, is an area rich in rER and sER. CRT is translated on this ER, enabling it to sequester exchangeable Ca^2+^ in the subapical zone (Lenartowski et al. 2015; Suwińska et al. 2015). Thus, we propose that in the absence of CRT, Ca^2+^ level remains high in the subapical domain; as a result, AFs are destroyed in this region.

Actin polymerization is necessary for secretory vesicles to accumulate in the apical inverted cone, but actin depolymerization is required for vesicle docking and fusion at the plasma membrane (see review by Staiger et al. 2010). In the lily, myosin XI is primarily localized at the apical domain, including the front end of the actin fringe, suggesting that this motor protein is responsible for carrying vesicles to the pollen tube apex (Lovy-Wheeler et al. 2007). It is also well known that the high level of Ca^2+^ at the tip is associated with secretion (Battery et al. 1999). Additionally, vesicle trafficking and docking within the clear zone of the growing pollen tube correlate directly with oscillatory changes in apical [Ca^2+^]_c_ (Comacho and Malhó 2003). Our data here show that knockdown of *PhCRT* in *Petunia* pollen tubes results in apical F-actin accumulation, disappearance of the tip-focused Ca^2+^ gradient, substantially fewer vesicles, and abnormal presence of residual CRT in the clear zone. Additionally, F-actin clusters were not present in highly vacuolated si1*PhCRT* tubes. Thus, we propose that CRT reduction in the subapical zone disrupts Ca^2+^ oscillations that are critical for exocytosis (and possibly membrane recycling) in the growing domain of the pollen tube. All these findings suggest that CRT indirectly affects the function of several ABPs, and thus pollen tube elongation, by regulating Ca^2+^ homeostasis in distinct pollen tube zones.

### CRT is crucial for ER structure, localization, and function in growing pollen tubes

One of the most obvious ultrastructural features of growing pollen tubes is the enrichment of rER and sER in the subapical zone (see reviews by Cheung and Wu 2007; Cai et al. 2015; Hepler and Winship 2015). We showed that this ER is co-localized with *PhCRT* mRNA, CRT protein, and rRNA in *Petunia* pollen tubes and concluded that CRT is translated on ER membrane-bound ribosomes at the sub-apex (Suwińska et al. 2015). Particularly, intense CRT labeling at the subapical zone has also been reported in vitro in *Nicotiana* pollen tubes (Nardi et al. 2006) and in vivo in *Petunia* and *Haemanthus* tubes penetrating the pistil (Lenartowska et al. 2002, 2009; Lenartowski et al. 2015). Thus, in addition to Ca^2+^ homeostasis, an important role of CRT immediately proximal to the growing domain of the tube may be regulation of chaperone activity in the quality control of glycoproteins passing through the ER. In fact, these two primary activities have been confirmed for animal and plant CRTs (see reviews by Jia et al. 2009; Michalak et al. 2009). Here, we show that knockdown of *PhCRT* in elongating pollen tubes strongly disturbed both ER localization and structure (Fig. 6). Reduction of CRT prevented ER accumulation in the sub-apex and may explain why ER instead was observed in the clear zone and occasionally the highly vacuolated shank. The ER cisternae in si1*PhCRT* pollen tubes were highly fragmented or disorganized, making distinction between rER and sER extremely difficult. In si1*PhCRT* pollen tubes, protein folding may be impaired, leading to the accumulation of unfolded proteins in the ER. These dysfunctional proteins may, in turn, disturb the ER ultrastructure. Given that extremely fast pollen tube growth requires very high rates of protein synthesis, such an effect of loss of CRT could contribute to abnormal tube elongation. Future studies will address this possibility.

Taken together, our data indicate that CRT is a key regulator involved in multiple aspects of *Petunia* pollen tube elongation: stabilization of Ca^2+^ homeostasis, regulation of the actin cytoskeleton arrangement/function, control of secretion, and maintenance of ultrastructure. We also suggest that CRT’s molecular chaperone and Ca^2+^-buffering activities facilitate elongation, a process that requires high rates of protein synthesis and precise regulation of Ca^2+^ level in distinct pollen tube zones. Our conclusion confirms the fact that *PhCRT* from *Petunia* is a highly conserved gene belonging to the *CRT1/CRT2* subfamily (Lenartowski et al. 2014), encoding the homolog that plays general roles associated with Ca^2+^ homeostasis and protein folding in plants (see review by Jia et al. 2009). Further characterization of CRT-dependent processes and the extent of cross-talk with other important pathways and molecules is now required to fully understand the molecular mechanism by which CRT acts to control pollen tube growth.

#### *Author contribution statement*

Conceived and designed the experiments: RL, ML. Performed the experiments: AS, PW, RL. Performed the quantitative analysis: RL, AS, PW, PZ. Analyzed the experimental and statistical data: RL, AS, PW, PZ, ML. Wrote the paper: RL, ML.

## Electronic supplementary material

Below is the link to the electronic supplementary material.

**Online resources S1** The average length and growth rate of *Petunia* WT (*n* ≥ 50) and si1*PhCRT* (*n* ≥ 50) elongating pollen tubes (TIFF 78757 kb)

## Abbreviations

ABP/s: Actin-binding protein/s
AF/s: Actin filament/s
*CRT/*CRT: Calreticulin gene/protein
CRT PAb: Polyclonal antibody against CRT
F(G)-actin: Filamentous (monomeric globular) actin
FISH: Fluorescent in situ hybridization
MGU: Male germ unit
siRNA: Small interfering RNA
si1*PhCRT*: si1RNA selectively degrading *PhCRT*
scr_si1*PhCRT*: Scrambled version of si1*PhCRT*
ssDNA: Single stranded DNA oligonucleotide
*PhCRT*: *Petunia hybrida CRT* gene
PTGS: Post-transcriptional gene silencing
